# Hourly Breast Expression to Estimate the Rate of Synthesis of Milk and Fat

**DOI:** 10.3390/nu10091144

**Published:** 2018-08-22

**Authors:** Jacqueline C. Kent, Hazel Gardner, Ching-Tat Lai, Peter E. Hartmann, Kevin Murray, Alethea Rea, Donna T. Geddes

**Affiliations:** 1 School of Molecular Sciences, The University of Western Australia, 35 Stirling Hwy, Crawley, WA 6009, Australia; Hazel.Gardner@uwa.edu.au (H.G.); Ching-Tat.Lai@uwa.edu.au (C.-T.L.); Peter.Hartmann@uwa.edu.au (P.E.H.); Donna.Geddes@uwa.edu.au (D.T.G.); 2 School of Population and Global Health, The University of Western Australia, 35 Stirling Hwy, Crawley, WA 6009, Australia; Kevin.Murray@uwa.edu.au; 3 Centre of Applied Statistics, The University of Western Australia, 35 Stirling Hwy, Crawley, WA 6009, Australia; Alethea.Rea@uwa.edu.au

**Keywords:** human lactation, expressing, milk synthesis, fat synthesis

## Abstract

Objective measurement of the rate of synthesis of breast milk and fat in breastfeeding mothers requires test-weighing of each breastfeed and the measurement of each expression from each breast over 24 h, with the collection of milk samples before and after each breastfeed and expression. We sought an abbreviated technique for measuring these rates of synthesis. Participants completed a 24-h breastfeeding milk profile, and expressed their breasts on arrival at the research room and each hour thereafter for 3 h (4 expressions). The hourly rate of milk synthesis, as measured by the yield of milk from the fourth expression, was closely related to the hourly rate of milk synthesis calculated from the 24-h milk profile. The hourly rate of fat synthesis, calculated from the fat content of small samples of the first and last milk expressed during the fourth expression, was different from the rate of fat synthesis calculated from the fat content and volumes of all the breastfeeds and expressions during the 24-h milk profile. The study confirms the use of an abbreviated technique to measure the rate of breast milk synthesis, but is not reliable as a measure of the rate of fat synthesis for an individual.

## 1. Introduction

Many mothers are not achieving the recommendations of the World Health Organization, the National Health and Medical Research Council, and the American Academy of Pediatrics of exclusive breastfeeding for 6 months [1,2,3,4,5]. Over 90% of mothers in Perth, Western Australia commence breastfeeding, but fewer than 1% are exclusively breastfeeding at 6 months [3]. In the United States 81% of mothers ever breastfed, and 22% are exclusively breastfeeding at 6 months [5]. A common reason given for early weaning is a perception of insufficient milk [6] that in turn is associated with a lack of confidence in breastfeeding [7]. Clinical indications of infants receiving enough breast milk include sufficient bowel movements and urine output [8] and satisfactory weight gains [9]. These are believed to be sufficient to provide reassurance for both parents and health professionals. However, the high frequency mothers who have a perception of insufficient milk indicates that further objective measures of milk production are required. 

Objective measurement of milk supply can be made by carrying out a 24-h milk profile that involves weighing the fully-clothed infant before and after every breastfeed at home and recording volumes of milk expressed (if applicable) for 24–26 h [10]. This allows calculation of both the total daily milk intake of the infant over 24 h, and the total daily milk production of each breast [10]. The average hourly rate of milk synthesis can then be calculated from the total milk production in 24 h divided by 24. If a mother is reluctant to measure her milk profile over a full 24 h, a small study has demonstrated that when the breasts are expressed using an electric breast pump each hour, the hourly rate of milk production from the 2nd to the 7th hour (the 3rd to the 8th expression) represents the longer-term physiological rate of milk synthesis [11]. The authors suggested that further studies are required to confirm this finding.

In addition to the volume of milk ingested by the infant, total caloric intake is important for energy expenditure and growth. Fat provides 49% of the energy in breast milk [12], and therefore the contribution of fat to the energy intake of the infant is significant. The daily fat intake of breastfed infants has been underestimated when unsuitable sampling regimes have been used. For example, calculations based on daily milk intake by test-weighing and fat content of milk samples collected before and after the first breastfeed of the morning [13] will underestimate the daily fat intake of the breastfed infant, because the fat content of milk in the morning is less than during the day and afternoon [14]. Another approach was to feed the infant from one breast only at each breastfeeding session and pump the other breast, alternating the breasts, for a 24-h period and measure the fat content of the pooled pumped milk [12]. The yield of milk from the pumping sessions averaged 49% of the 24-h milk production determined by test-weighing, but some were <40% and others >60% [12], indicating that this technique may be sufficiently accurate for a population study, but not useful to predict the daily fat intake for an individual infant. 

When mothers collect small samples (<1 mL) of breast milk before and after each breastfeed during the 24-h milk profile, the total fat intake can be calculated from the fat content of the samples and the volume of each breastfeed. If the mother is also expressing breast milk, the measurement of the fat content of small samples (<1 mL) of breast milk collected before and after each expression and the volume of each expression, in addition to the data from the 24-h breastfeeds, allows calculation of the mother’s total daily fat production. The hourly rate of fat synthesis can then be calculated by dividing the total fat production in 24 h by 24.

We aimed to provide further data to investigate if the technique of hourly pumping can be used to estimate the rate of milk synthesis, and ascertain if the same technique could be used to estimate the rate of milk fat synthesis if mothers prefer this technique to 24-h test-weighing with milk samples.

## 2. Materials and Methods

We recruited lactating mothers between 5 and 11 months from birth of term singleton infants who agreed to measure their 24-h milk profile with milk samples, and come to the research room at The University of Western Australia for a three-and-a-half-hour study session. Participants were only included in the study if their infants had previously accepted breast milk from a bottle. The study was approved by the Human Research Ethics Committee of The University of Western Australia (RA/4/1/4492) and all participants provided written informed consent. 

Demographics were recorded and the participants were loaned accurate digital scales (BabyWeigh^™^, Medela Inc., McHenry, IL, USA, resolution 2 g, accuracy ± 0.034%) to measure their 24-h milk profile. All measurements of breastfeed volumes and milk production were made in grams but expressed in mL because the density of milk is 1.03 g/mL [15]. Data were recorded either on paper or entered on a secure password-protected website accessed by invitation only. The corrected 24-h milk production, for participants who were either exclusively breastfeeding or breastfeeding and expressing, was calculated by the method of Arthur et al. [10]. However, no correction for infant insensible water loss was made, and therefore milk production may be underestimated by an average of 10% (range 3–55%) [10]. The mean rate of milk production for each breast was calculated by dividing the corrected 24-h milk production for each breast by 24.

During this 24-h period, the mothers hand-expressed small milk samples (<1 mL) into 5-mL polypropylene plastic vials (Disposable Products, Adelaide, SA, Australia), immediately before and after each breastfeed or expression from each breast. Samples were frozen as soon as possible and kept at -15°C until analyzed. The cream content was measured using the creamatocrit method [16]. The cream content of the milk of each feed or expression during the measurement of milk profile was calculated as ([0.53 × pre-feed creamatocrit + 0.47 × post-feed creamatocrit]/2) [17]. The creamatocrit was converted to fat using the following formula: fat (g/L) = 3.968 + (5.917 × creamatocrit) [16]. The amount of fat in each breastfeed or expression was calculated from the fat content of the milk and the volume of the feed or expression. The total amount of fat synthesized by each breast in the 24-h period was calculated by summing amount of fat in all the breastfeeds and expressions from that breast, and the mean rate of fat synthesis was calculated by dividing the total amount of fat synthesized for each breast by 24.

For the hourly-pumping study the participants pumped either both breasts simultaneously or one breast only according to the preference of the participant. On arrival at the research room at The University of Western Australia, the mothers expressed their breast(s) using a Medela Symphony breast pump at their own maximum comfortable vacuum for 10 min after milk ejection was detected by an increase in milk flow. For each breast, milk was conveyed via a connecting tube from the breast shield to one of three bottles placed on the weigh platform of a continuous weigh balance (ShowMilk, Carag AG, Baar, Switzerland). The first 1 mL (first milk) was collected into the first bottle, the bulk of the expressed milk was collected into the second bottle (pooled milk), and the last milk expressed (~1 mL) was collected into the third bottle (last milk). The ShowMilk device measures the cumulative weight of milk at 50 Hz with a resolution of 0.1 g and accuracy 0.02% to a maximum of 2 kg [18]. The expression was repeated 1, 2 and 3 h after the commencement of the first expression. The total volume of each expression (mL) from each breast was recorded. 

The creamatocrit of the first, pooled, and last milk of each expression was measured as above and converted to fat content (g/L) [16]. This, with the total volume of milk expressed, was used to calculate the total amount of fat in the expressed milk for each hour.

From the 24-h milk profile, the fat content of all the milk samples and the volumes of all the breastfeeds were used to estimate the breastfeeding storage capacity of the breasts [19]. If the participants expressed their breast milk on one or more occasions during the 24 h, the data from the expressions were included with the breastfeeding data to calculate the potential storage capacity of the breasts [20]. The fat content of the first milk samples was used to calculate the degree of fullness of the breast before the expression [21].

### Statistics

Data are presented as mean ± SD or median (IQR). A paired *t*-test was used to compare fat content of first milk and last milk for each expression, and unpaired *t*-test was used to compare total milk production of participants who were exclusively breastfeeding with those who were breastfeeding and expressing. Linear mixed models were used to analyse the responses volume of fourth expression, rate of synthesis, amount of fat fourth expression, and rate of fat synthesis using a fixed effect of method (hourly pumping vs. 24-h profile) and random effects of participant and breast within participant. The volume of each expression, and the fat content of the first and last milk at time points 0, 1, 2, and 3 h of the hourly pumping study were included in the fixed effect of method. Key comparisons were considered using general linear hypothesis tests (with a Tukeys adjustment). Significance was set at the 5% level, and data were analysed using the R environment for statistical computing [22].

## 3. Results

Data were collected from 15 participants, of whom 10 were feeding male infants and 5 female infants. A 24-h milk profile was measured by 11 participants on a day during which they were exclusively breastfeeding and 4 participants on a day during which they were breastfeeding and expressing (1 participant on two occasions). The demographics of the participants are presented in Table 1. A single study session occurred within 11 weeks of the measurement of 24-h milk profile for 14 of the participants. One participant measured her 24-h milk profile when her infant was 5 weeks old and participated in a study session 6 weeks later. She measured her 24-h milk profile again when her infant was 17 weeks old and participated in a second study session 4 weeks later. During the study sessions 3 participants expressed from one breast, 11 expressed from both breasts, and 1 expressed from both breasts for 2 study sessions, providing data for 29 breasts. Milk samples before and after each breastfeeding or expression during the 24-h milk profile were collected by 11 of the participants, and 4 participants did not collect milk samples. The breastfeeding storage capacity [20] (calculated from the fat content of samples collected before and after breastfeeds only) was 165 ± 42 mL. When the fat content of all samples from all breastfeeds and expressions, including those from the study sessions, was included the potential storage capacity [20] was 182 ± 58 mL. On average, the breastfeeding infants took 67 ± 11% of the milk that was available in the breast when the 24-h milk profile was measured. 

There was no significant difference in 24-h milk production between participants who were exclusively breastfeeding and those who were breastfeeding and expressing (difference = 68.6 mL, 95% CI: 101.9–239.1, *p* = 0.400)

**Figure 1:**
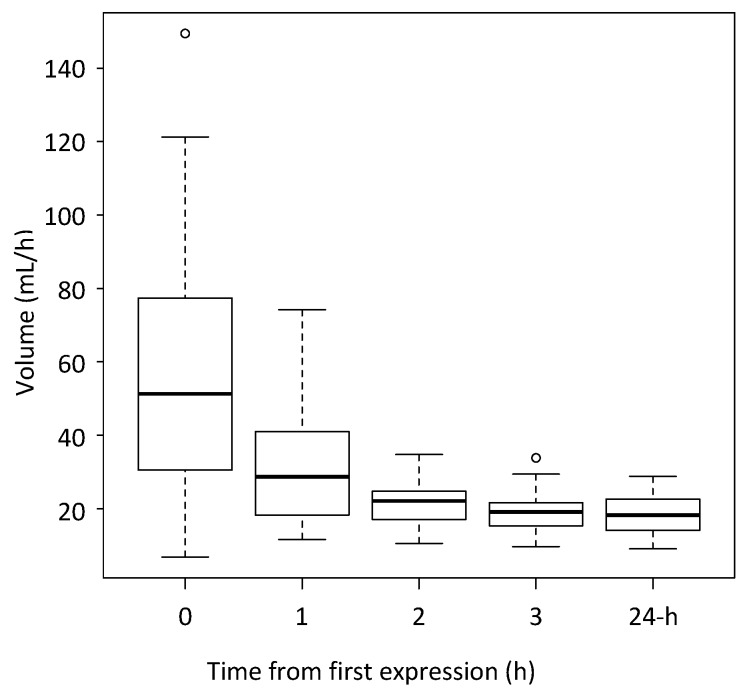
The yield of milk from hourly expressions, and the hourly rate of milk synthesis calculated from the 24-h milk profile data (24-h). *n* = 29 breasts.

The yield of milk from each expression during the study session and the hourly rate of milk synthesis calculated from the 24-h milk profile data are presented in Figure 1.

On average the yield of milk from each of the second, third and fourth expressions (1, 2 and 3 h after the first expression) was lower than for the expression the hour before (mean expression 59.0 mL at time 0, 32.0 mL at time 1, 22.0 mL at time 2 and 18.9 mL at time 3). 

**Figure 2:**
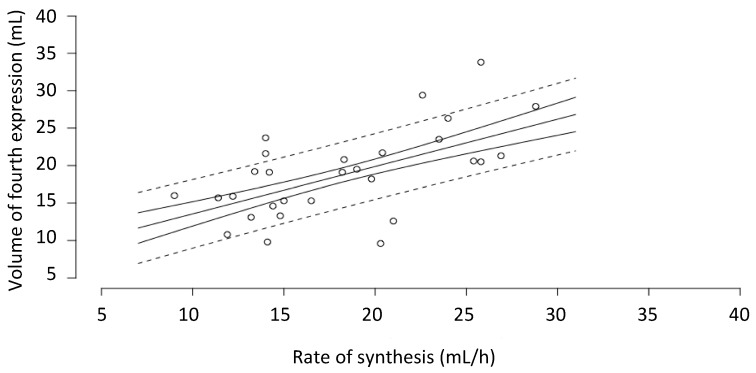
Relationship between the volume of milk from the fourth expression (3 h after the first expression) and the hourly rate of milk synthesis calculated from the 24-h milk profile data. Fitted model (solid line), with the confidence (solid line) and prediction intervals (dashed lines). *n* = 29 breasts.

The yield of milk from the fourth expression (3 h after the first expression) (18.9 ± 5.8 mL) was not significantly different from the hourly rate of milk synthesis (18.2 ± 5.4 mL/h) calculated from the 24-h milk profile data (mean difference = 0.966, SE = 0.995, *p* = 0.997). There was also a statistically significant relationship between the volume of the fourth expression and rate of synthesis (*p* = 0.002) (Figure 2).

**Figure 3:**
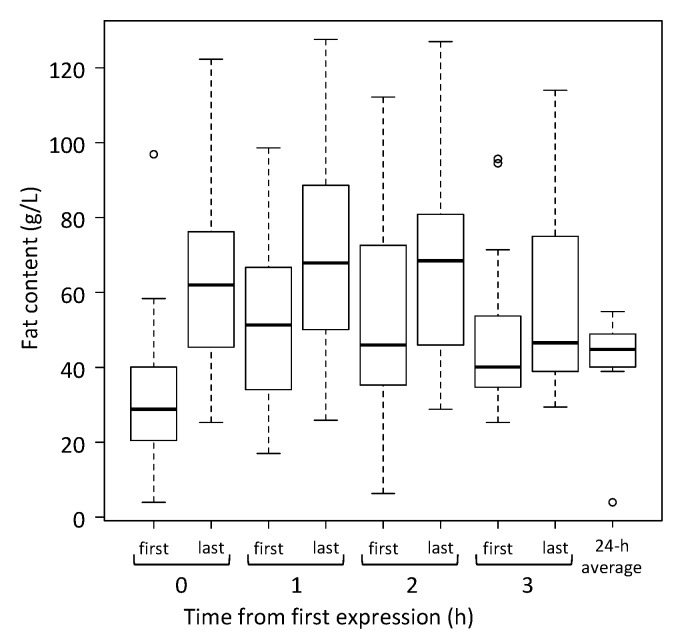
The fat content of first and last milk collected at each expression and the average fat content of milk samples collected during the 24-h milk profile data (24-h average). *n* = 29 breasts.

The fat content of the first and last milk collected at each expression and the average fat content of milk samples collected during the 24-h milk profile are presented in Figure 3. The fat content of the last milk was higher than the first milk for each expression but not statistically different at time 3 (time 0: mean difference = 34.9 g/L, SE = 8.1, *p* = 0.001; time 1: mean difference = 22.0 g/L, SE = 8.1, *p* < 0.001; time 2: mean difference = 17.8 g/L, SE = 8.1, *p* = 0.021; time 3: mean difference = 15.7 g/L, SE = 8.1, *p* = 0.092). There was no significant difference in the fat content of milk collected at the end of each of the first, second, third or fourth expressions (63.7 ± 27.6 g/L, 69.1 ± 28.2 g/L, 69.1 ± 29.4 g/L, 58.4 ± 27.0 g/L respectively). There was no significant difference between the fat content of pooled milk from the fourth expression (52.5 ± 23.5 g/L) and the average fat content of milk collected over the 24-h period (46.6 ± 8.1 g/L) (mean difference = 10.5 g/L, 95% CI: 7.2–20.1, *p* = 0.18).

**Figure 4:**
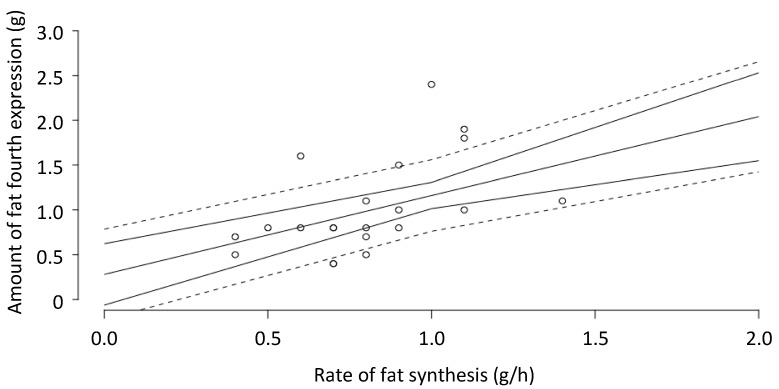
Relationship between the amount of fat from the fourth expression and the hourly rate of milk fat synthesis calculated from the 24-h milk profile data (*p* = 0.054). Fitted model (solid line), with the confidence (solid line) and prediction interval (dashed line). *n* = 29 breasts.

The amount of fat in the milk from the fourth expression was 1.02 ± 0.50 g. This was different from the hourly rate of fat synthesis (0.79 ± 0.23 g/h) calculated from the 24-h milk profile (mean difference = 0.23 g/h, SE = 0.10, *p* = 0.034). There was no significant relationship between the two measurements (*p* = 0.054) (Figure 4).

The degree of fullness of the breast before the first expression (time 0), calculated from the fat content of the first milk and the potential storage capacity of the breast, was 0.6 ± 0.2. The first expression removed 53 ± 26% of the available milk, with 6 sessions classified as low outcome (<40% of the available milk removed), and 5 sessions classified as high outcome (>70% of the available milk removed). 

## 4. Discussion

The data suggest that all the milk from the fourth expression had been synthesized since the end of the third expression, and the yield of milk from that hour (50 minutes after the last expression plus 10 minutes expressing) was closely related to the hourly rate of milk synthesis calculated from the 24-h milk profile data. Therefore, this study has confirmed the findings of Lai et al. [11] that for mothers who are fully breastfeeding the technique of hourly pumping for 4 expressions can be used as a measure of the hourly rate of milk synthesis. It was established by Lai et al. that hourly pumping for a further 3 h yielded the same volume as the fourth expression, indicating that the steady state of milk synthesis and removal had been reached, and more than three hourly expressions after the first expression are not required. It has been established that an accurate measure of 24-h breast milk production cannot be calculated by test-weighing an infant for a 12-h period (6 a.m. to 6 p.m.) and doubling the total [14,23], nor by taking the mean milk intake for two consecutive feedings and multiplying it by the number of feedings in the 24-h period [23]. If mothers are reluctant to weigh their infants before and after every breastfeed for a full 24 h, or if they urgently need to know their rate of breast milk synthesis and are prepared to express their breasts every hour for 3 h after the first expression, this technique can provide reliable information. If milk production is less than optimal, early intervention is beneficial [24]. An objective measurement of the rate of milk synthesis can guide advice from a clinician on increasing milk production if required. It must be borne in mind that the infant will probably need to be given milk by another means unless there is usually an interval between breastfeeds of more than 3 h. 

The proportion of mothers in Australia who combine breastfeeding and expressing is increasing [25], either to leave expressed breast milk with a carer, or to increase milk supply [26]. Mothers with concerns about their infants’ transfer of milk from the breast need to undertake a full 24-h period of test-weighing to determine the efficacy of their breastfeeding. Using the hourly pumping technique will provide information about their current total rate of milk production, and can be used to monitor the effects of alterations in their expression regime to increase milk production. 

The finding that the milk removed during the fourth expression represents the hourly rate of milk secretion suggests that the yield of fat should represent the hourly rate of fat secretion. Although there was a trend towards a relationship between the amount of fat in the last expression and the hourly rate of fat synthesis calculated from the 24-h profile data, the mean difference was 0.23 (a reduction of 23%), indicating that this technique is not reliable for predicting the rate of fat synthesis for an individual mother. 

The changes in fat content before and after each expression are interesting, and an explanation is worthwhile. The breast reaches its maximum degree of fullness following the longest interval after the previous removal of milk. This long interval allows time for the fat globules in the alveoli to partition, as they tend to adhere to the surface of the lactocytes, resulting in the first milk expressed being low in fat content [27]. The wide range in fat content before the first expression is a result of both the variable time since the previous removal of milk from the breast and inter-individual differences in fat content of milk [28]. The fat content increases as milk is removed from the breast, reaching a maximum when the breast is fully drained [27,29]. The wide range in fat content after the first expression is a result of the variability between mothers and the effectiveness of milk removal by a breast pump [18]. In the 50 min between the end of the first expression and the start of the second expression, the fat content of the milk within the alveoli (69.1 g/L) was diluted by the newly-secreted milk with an average fat content of (41.1 g/L) [14] to reach 51.3 g/L (Figure 3). Compared with the previous interval, there was less time for the fat globules to partition between the lumen of the alveoli and the luminal wall of the lactocytes, resulting in a smaller difference between the first and last milk of the second expression (Figure 3). The higher fat content of the last milk of the second expression occurred because the first expression did not drain the breast completely (53% of the available milk removed), leaving milk with a high fat content in the alveoli. The wide variability is the result of the 6 sessions that were low outcome (<40% of the available milk removed). The yield of milk from the third expression was significantly higher than from the fourth expression, indicating that the third expression did not quite drain the breast, and only the fourth expression comprised only milk secreted in the previous hour. This is consistent with the fat content of the last milk from the third expression remaining elevated (Figure 3). There was a small difference in the fat content between the first and last milk from the fourth expression, which was consistent with some partitioning of the fat globules during the 50 min after the end of the third expression.

It is puzzling that, although the volume of the last expression was closely related to the hourly rate of milk synthesis calculated from the 24-h milk profile data, the amount of fat in the last expression was not an accurate reflection of the rate of fat synthesis for an individual. We suggest that it is the result of the steep rise in fat content as the breast is drained [29,30] such that the final small volume of milk expressed makes a disproportionately large contribution to the fat content of the final milk sample.

The higher fat content of the second expression could make it suitable for feeding low-birth-weight infants when the mother has an abundant supply, with the milk from the first expression being stored for later use. This may be an alternative to fractionating an expression [31].

## 5. Conclusions

These data confirm that the fourth hourly expression can be used to estimate the average rate of breast milk synthesis for individuals and may be useful when low milk production is suspected. However, while there is a tendency to an overall relationship between hourly pumping and 24-h milk profile for estimating the rate of synthesis of milk fat, this technique is less accurate for individuals.

## Figures and Tables

**Table 1:** Demographics of participants (*n* = 15).

	Median (IQR)
**Infant**	
Age (weeks)	22 (12)
Birth weight (g)	3650 (726)
Fat intake (g/24h) (*n* = 11)	34 (13)
**Mother**	
Age (years)	35 (37)
Exclusive breastfeeding during 24-h milk profile measurement (*n* = 11)	
24-h milk production (mL)	860 (237)
Breastfeeding and expressing during 24-h milk profile measurement (*n* = 5 *)	
Milk transfer breastfeeding (breast to infant) (mL)	565 (311)
Milk transfer (breast to bottle) (mL)	180 (124)
Total 24-h milk production (mL)	810 (224)

* 1 participant measured her 24-h milk profile on 2 occasions.
